# Rapid method to identify the boundary speed for metabolic stability from race performances

**DOI:** 10.1080/11791543.2026.2613468

**Published:** 2026-01-30

**Authors:** Samuel N. Cheuvront, Jamie Ferris, Ben Hazelwood, Shawn E. Bearden, Jinger S. Gottschall

**Affiliations:** a New Balance Sports Research Lab, Brighton, MA, United States; b Idaho State University, Pocatello, ID, United States

**Keywords:** VDOT, running economy, running performance, critical speed, maximal metabolic steady state

## Abstract

Develop and validate a rapid method for estimating metabolic stability from race performances. Nineteen runners (12 male, 7 female) provided ≥1 race times for distances from 1.5 to 42 km. Race times were used to estimate the boundary speed for metabolic stability (MS_lim_) from six regression equations. Twelve males ran for 5-min at a self-selected speed between 60−90% of the estimated MS_lim_. Seven females ran for 5-min at 90% and 110% of estimated MS_lim_. Oxygen uptake (V̇O2) was measured and the slope of the line describing the final two minutes of gas sampling was used to quantify metabolic stability. Runners self-selecting speed (79 ± 10% of estimated MS_lim_) exhibited metabolic stability (zero slope; *p =* 0.88) with a mean 2-min change in V̇O2 of 35 ± 28 mL/min. Runners at 90% of estimated MS_lim_ also exhibited metabolic stability (zero slope; *p =* 0.31) with a mean 2-min change in V̇O2 of 66 ± 43 mL/min. Respiratory exchange ratios were <0.90, also consistent with steady state metabolism. Metabolic stability was not possible at 110% of MS_lim_. A rapid method for estimating the boundary speed for MS_lim_ from race performances is provided for when more extensive metabolic testing is not possible.

## Introduction

Treadmill speed selection can be a quandary for sports science researchers, particularly when time and resources are limited. Treadmill speed is customarily individualised relative to specific metabolic boundaries, beyond which physiological homoeostasis is challenged (Jamnick et al., 2020). Speeds below the upper limit of metabolic stability (MS_lim_) permit a metabolic steady state, reflecting an intramuscular balance between oxygen supply and demand (Balasekaran et al., 2023; Goulding et al., 2021; Grassi et al., 2011; Poole et al., 2016). Treadmill speeds that produce metabolic stability are critical when prescribing moderate to vigorous intensity physical activity (MacIntosh et al., 2021; Wolpern et al., 2015), when assessing performance potential (Balasekaran et al., 2023), or when testing running economy, defined as the steady state oxygen or energy demand of running (Daniels, 1985).

Metabolic stability can be estimated using measurements of gas exchange, lactate metabolism, or critical speed (CS) testing (Jones et al., 2019), all of which require multiple lab visits and often exhaustive exercise bouts (Balasekaran et al., 2023; Matthews et al., 2023). Simple solutions for estimating the treadmill boundary for CS include variations of the talk test (Reed & Pipe, 2014), or the use of multiple race times. The latter affords greater precision but traditionally requires three race distances between 2- and 20-minutes duration to estimate CS (Jones et al., 2019; Pettitt, 2016). Solutions using single race speeds include 5 km race speed (Pettitt et al., 2013) or 10 km race speed minus 1 km/h (Padulo et al., 2012). To our knowledge, an expedient method flexible enough to estimate metabolic stability from any race finishing times has not been proposed. Jack Daniels’ running tables are used by nearly 500,000 runners and their coaches (Daniels, 2014) in a variety of ways, one of which is to estimate current fitness levels (colloquially VDOT, or V̇O_2_max) from recent running performances over popular race distances (Scudamore et al., 2018). The tables also permit extrapolation of race performances from one distance to another, across intensity domains, based upon predictable physiological relationships (Daniels, 1979). We reasoned that the performance times in these tables will predict the CS for runners of any ability using traditional methods for estimating CS (Jones et al., 2019; Pettitt, 2016). From these relations we should be able to derive novel, expedient regression equations that identify the boundary of metabolic stability (V̇O_2_ steady state) from any runner’s real–world race time(s).

The development of an expedient and widely applicable tool, one that can translate a single race performance over a broad range of distances into an estimate of metabolically stable treadmill speed, could fill a meaningful gap in applied exercise physiology (Balasekaran et al., 2023; MacIntosh et al., 2021; Matthews et al., 2023; Wolpern et al., 2015). Such a tool could enhance the feasibility of training prescription, improve standardisation in research protocols, and reduce both time demands and participant burden. Ultimately, a simple race–time–based estimator of treadmill metabolic stability could empower sport scientists, clinicians, and coaches to obtain a first approximation of more empirical metabolic testing results in circumstances where comprehensive testing is impractical or impossible.

The purpose of our study was to develop predictive equations that use any recent track or road race times and test the hypothesis that they will identify the boundary between V̇O_2_ steady state and non–steady state (i.e., the limit of metabolic stability, MS_lim_).

## Methods

### Participants

Two groups of runners participated in this study. Twelve trained (McKay et al., 2022) male runners (age: 27 ± 5 years, height: 175±3 cm, weight: 69.1±7.3 kg) and seven highly trained (McKay et al., 2022) female collegiate track runners (age: 20 ± 2 years, height: 170 ± 4 cm, weight: 59.1 ± 5.2 kg) provided recent race times (within past 12 months). Trained runners provided recent times for races of 5, 10, 21, and 42 km; highly trained runners provided times for 1.5, 3, and 5 km. Six runners provided a time for 1.5 km (4:16−4:35), n = 5 for 3 km (9:12−9:50), n = 12 for 5 km (15:50 to 22:43), n = 6 for 10 km (32:31 to 46:12), n = 7 for 21 km (1:11:23 to 1:43:00), and n = 3 for 42 km (2:31:40 to 3:40:00). All runners were free of pain when running and were >6 months post any lower limb injury. All runners had at least 2 years of training/racing experience. For trained runners, weekly distance ranged from 16−32 km/week to 80+ km/week, frequency of runs ranged from 1−3 days per week to 6+ days per week, and typical run paces ranged from 9.65 km/h to 14 km/h. Training habits of the highly trained runners were unknown but were consistent with division 1 runners at the start of their cross country season. No restrictions were placed on the trained runners; highly trained runners were asked to prepare for the training session as they would for a 5 km race. Testing occurred in September 2024 (males) or September 2025 (females). Males wore a test shoe similar to the New Balance Fuel Cell SC Elite v4; females wore their own preferred training or racing footwear. All runners wore their own preferred clothing.

### Protocol

Jack Daniels’ running tables (Daniels, 1979) were refined to n = 6 columns of running distances (1.5, 3, 5, 10, 21, 42 km) and n = 46 VDOT rows (i.e., V̇O_2_max) ranging from 40 to 85 ml/kg/min. Although Daniels included many more distances, we chose those raced most on the track and road. Our VDOT range is also smaller so that when choosing running speeds at the lower bound of metabolic stability, a value of 60% corresponds to a minimum running speed of 7.2 km/h (i.e., MS_lim_ ~12 km/h). VDOT increments were set to 1 ml/kg/min (rather than 0.1 ml/kg/min) (Daniels, 1979) to avoid artificial precision.

CS was first calculated for each VDOT row using 1.5, 3, and 5 km distances and finishing times from the table in accordance with the formula, D = CS^∗^T + D’, whereby the slope of the regression line relating distance (D, km) to time (T, hours) returns CS (km/h) (Jones et al., 2019; Pettitt, 2016). The single column of CS was then uniquely regressed against each column of race times and converted to km/h to generate six linear prediction equations (Table 1), one for each race distance (1.5, 3, 5, 10, 21, 42 km). The MS_lim_ was then calculated for each study participant using the equations in Table 1 with individual race times as the ‘x’ variable input. When >1 race time was provided, MS_lim_ estimates were averaged. MS_lim_ was calculated (Table 1) for n = 9 participants using a single race time, n = 3 using two race times, n = 4 using three race times, and n = 3 using four race times.

Importantly, our experimental approach was to estimate running speeds that reliably produce metabolic stability (<MS_lim_) using real race times as a proxy. Our use of VDOT tables to first estimate CS lacks the precision of formally testing for CS (Hughson et al., 1984), but a precise CS prediction was not our intent in Table 1. Rather, we used VDOT tables to estimate CS as one of several possible threshold boundaries (Jones et al., 2019; Matthews et al., 2023) for metabolic stability, then applied our regression equations to estimate MS_lim_ and test for a metabolic steady state using treadmill speeds above (110%) and below (60−90%) it, as recommended for this purpose (Lansley et al., 2011). This produced a comprehensive test of our hypothesis, providing data throughout the heavy and into the severe intensity domains (Poole et al., 2016) against which <MS_lim_ was defined as metabolic stability and assessed by steady state V̇O_2_ measurement.

**Table 1.** Linear regression equations for estimating MS_lim_ from known race event speed.

Race event distance (km)	Linear Equation (km/h)
1.5	MS_lim_ (km/h) = 0.8352x + 0.4968
3.0	MS_lim_ (km/h) = 0.8913x + 0.5335
5.0	MS_lim_ (km/h) = 0.9487x + 0.1566
10.0	MS_lim_ (km/h) = 0.9939x – 0.0058
21.0	MS_lim_ (km/h) = 1.0310x + 0.2348
42.0	MS_lim_ (km/h) = 1.0950x – 0.0414

Where ‘x’ is race distance (km) divided by race time (h) = race speed (km/h); n = 46 data pairs per equation (R2 ≥0.99; SE = 0.01 to 0.06).

Gas exchange variables were measured using 20-s time averaging (Robergs et al., 2010) with a ParvoMedics metabolic cart (ParvoMedics TrueOne®, Salt Lake City, UT, USA). Total dead space of the system (mouthpiece, valve, collection tube, pneumotach, mixing chamber, and sampling tube) was 4.98 liters. The system was calibrated for flow (model 5530, Hans Rudolph, Inc., USA) and known gases (16% O_2_ and 4% CO_2_) before each test. All bouts of treadmill running were performed on a stiff deck instrumented treadmill (Treadmetrix, Park City, UT, USA).

Runners performed a 10-minute warm–up, running at any desired speed below 90% of the MS_lim_ estimate, followed by a five–minute rest. For trained runners, testing involved 5-minutes of treadmill running at a self–selected speed that was between 60 and 90% of the MS_lim_ estimate. For highly trained runners, testing involved two 5-minute bouts of treadmill running with a 5-minute rest in between, one at 90% and one at 110% of MS_lim_. The final 2-minutes of running were used to analyse the slope of the V̇O_2_ response. Environmental laboratory conditions ranged narrowly from 20−22^◦^C, 40−60% rh. Each participant was informed of the experimental procedures and associated risks before providing written informed consent. The study was approved by the BRANY Institutional Review Board (protocol #1402) and was therefore performed in accordance with the ethical standards in the Declaration of Helsinki.

### Statistical analysis

Descriptive data are reported as mean ± SD or (range). Where appropriate, tests for normality and skewness were performed using the D’Agostino–Pearson omnibus test. V̇O_2_ (L/min) sample averages from running minutes 4 and 5 were analysed using linear regression (n = 6 mean data points per participant). Group slope values not significantly different from zero (*p* > 0.05 = zero slope) were considered quantitatively synonymous with metabolic stability (i.e., a plateau in V̇O_2_ kinetics without evidence of a slow component). A slope coefficient between 0.025 and 0.075 was considered qualitative evidence of metabolic stability, assuming steady state V̇O_2_ is 2.5 L/min (i.e., ΔV̇O_2_ = 50 to 150 mL/min). A sample size of 8-12 participants provided sufficient power to detect a large effect size between 0.8 and 1.0, assuming conventional alpha (0.05) and beta (0.20) for a one sample *t*-test (n = [(Z_α_-Z_β_)σ/Δ]^2^). (Dawson-Saunders & Trapp, 1994). Statistical analyses were performed, and graphical displays were created using GraphPad Prism version 10.6.1 (GraphPad Software, La Jolla, CA, USA, www.graphpad.com).

## Results

**Figure 1.**
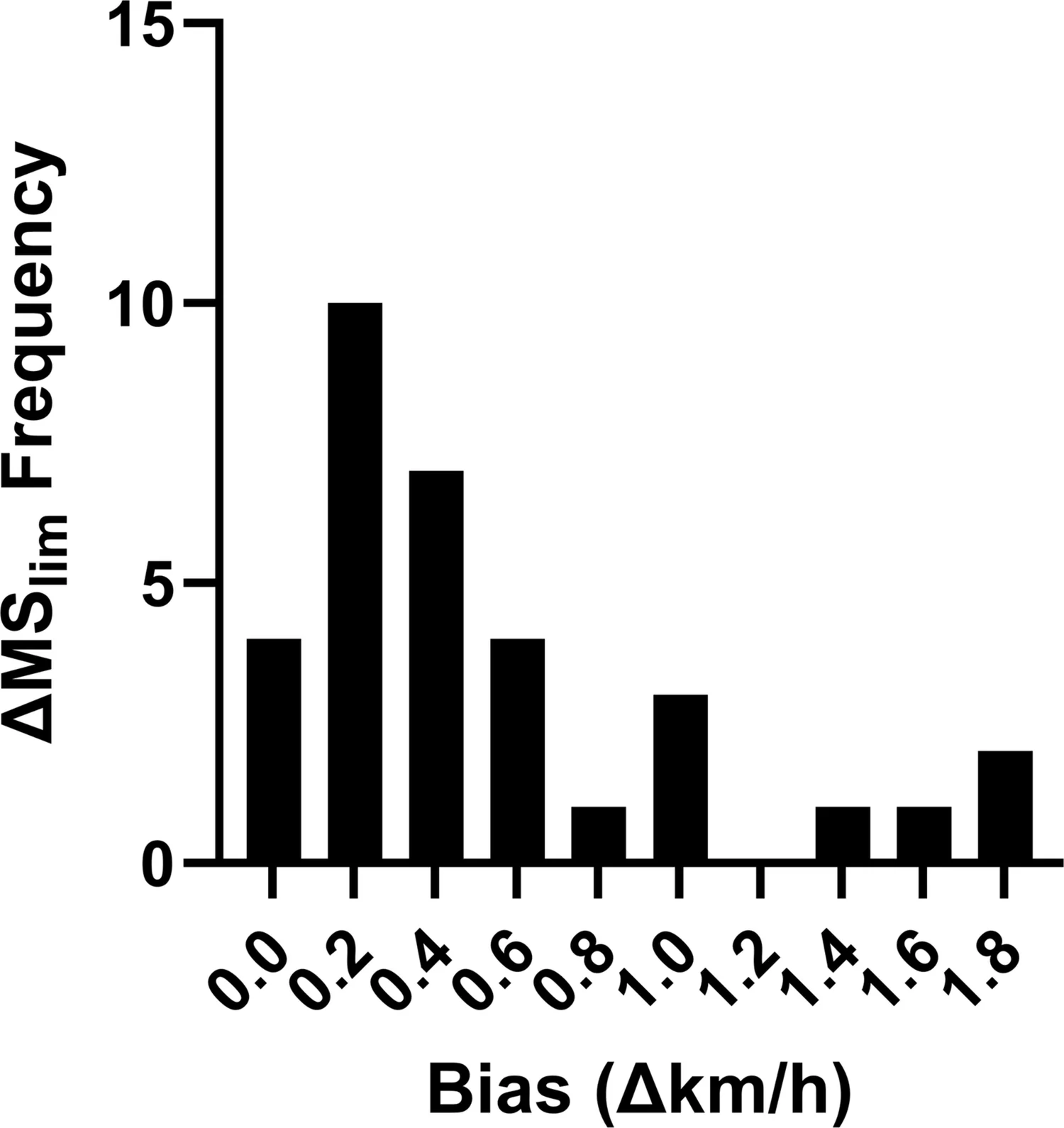
Frequency distribution of the absolute within–person bias (x–axis) among Table 1 MS_lim_ estimates from multiple race times. A total of 33 pairs of estimates were compared (y–axis); 1.5 vs 3 km (n = 3), 1.5 vs 5 km (n = 2), 3 vs 5 km (n = 2), 5 vs 10 km (n = 6), 5 vs 21 km (n = 6), 5 vs 42 km (n = 3), 10 vs 21 km (n = 5), 10 vs 42 km (n = 3), 21 vs 42 km (n = 3). Mean absolute bias was 0.54 km/h.

For the n = 10 runners who provided >1 race time, the absolute mean difference in the MS_lim_ estimates (n = 33 pairs of differences) from the regression equations in Table 1 was 0.54 km/h, or <5% for MS_lim_ above 12 km/h (Figure 1). Collectively, differences among the MS_lim_ estimates failed Gaussian assessment (*p* < 0.01), with most of the differences clustering at 0.20 km/h, creating a rightward skew in the distribution (Figure 1). Since MS_lim_ estimates from ≥2 race times were averaged, the mean absolute effective error for 10 of the 19 runners was half (i.e., 0.27 km/h).

**Figure 2.**
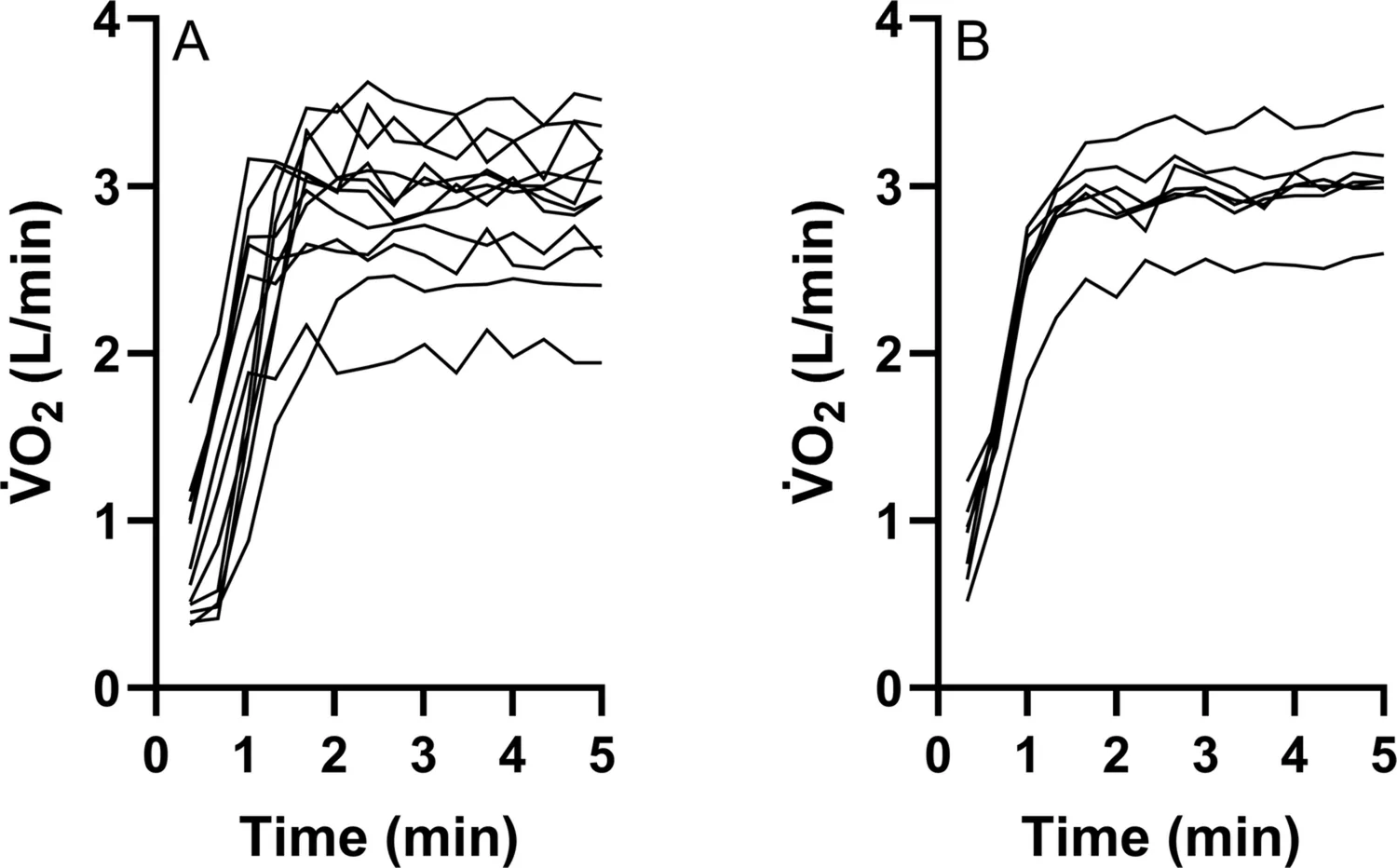
Individual V̇O_2_ uptake responses (0 to 5 minutes) for n = 12 trained male runners (A) and n = 7 highly trained collegiate runners (B) at 79 ± 10% and 90% of Table 1 MS_lim_ estimates, respectively.

**Figure 3.**
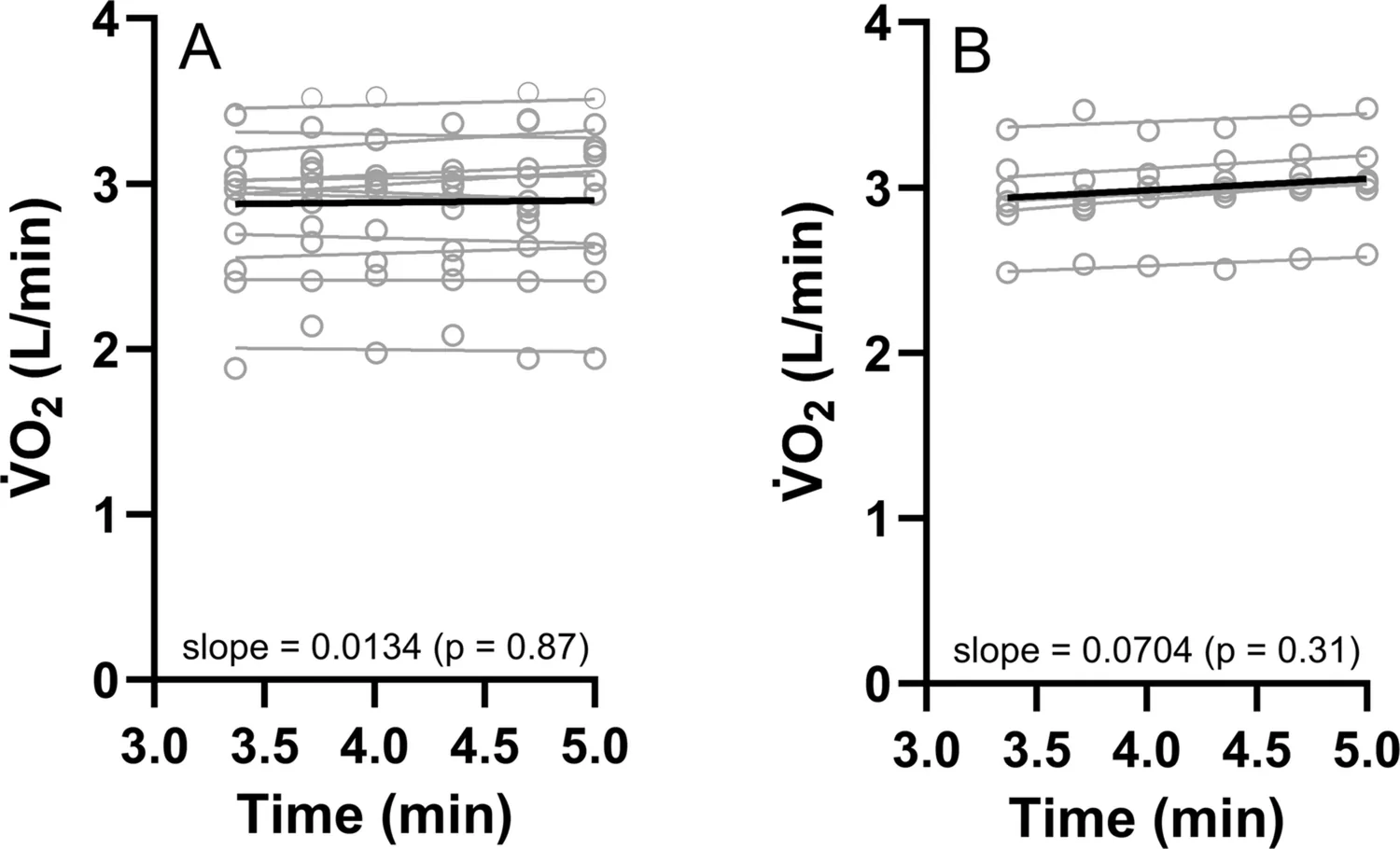
Individual (grey lines with circles) and group (black line) slope calculations for n = 12 trained male runners (A) and n = 7 highly trained collegiate runners (B) at 79 ± 10% and 90% of Table 1 MS_lim_ estimates, respectively.

The mean self–selected treadmill speed within the trained runner group was 12.3 ± 1.4 km/h, which was 79 ± 10% of the MS_lim_ speed estimate (15.6 ± 2.1 km/h). The ΔV̇O_2_ between running minutes 4 and 5 was 34.5 ± 27.7 mL/min (6 to 92 mL/min) with a slope not significantly different (*p =* 0.877) from zero (Figures 2A and 3A). For the highly trained collegiate female runners, speeds were set 10% below (15.64 ± 0.34 km/h) and above (19.11 ± 0.42 km/h) the MS_lim_ estimate from Table 1 (17.38 ± 0.38 km/h). The ΔV̇O_2_ between running minutes 4 and 5 at 90% of the MS_lim_ estimate was 66.1 ± 43.2 mL/min (3 to 115 mL/min) with a slope not significantly different (*p =* 0.310) from zero (Figures 2B and 3B).

The respiratory exchange ratios for the men (0.87 ± 0.04) and women (0.89 ± 0.04) were also consistent with submaximal, steady state metabolism (Wasserman et al., 1994) when speeds fell between 60 and 90% of the MS_lim_ estimate. However, all n = 7 collegiate runners fatigued within 3.5 minutes at 110% of the MS_lim_ estimate, thus no slope testing occurred, and it was concluded that running speed was > MS_lim_ and beyond metabolic stability.

## Discussion

The purpose of our study was to develop predictive equations that use recent track or road race times and test the hypothesis that they will identify the boundary between V̇O_2_ steady state and non–steady state (i.e., the point of metabolic stability, MS_lim_). We approached the problem by using Daniels’ VDOT running performance tables (Daniels, 1979, 2014) to generate novel regression equations which rely on race times to estimate MS_lim_. Our results support the use of this expedient method for selecting treadmill speeds that reflect metabolic stability, making the approach potentially useful any time the careful selection of treadmill speed is desired, but time and resources are limited.

Metabolic stability was achieved with treadmill speeds set to ≤90% of Table 1 MS_lim_ estimates (Figures 2A/B and 3A/B), independent of the race distance used to generate the estimate. This is an important observation supporting the physiological underpinnings of the VDOT tables, which are based on thousands of athlete observations (Daniels, 1979), and their consistency with classical energetics principles. Although simple suggestions for estimating a metabolic stability threshold from a single running distance, such as velocity at 5 km (Pettitt et al., 2013) or velocity at 10 km minus 1 km/h (Padulo et al., 2012, have been proposed, our equations (Table 1) include both distances while expanding race options both downward (1.5 and 3 km) and upward (21 and 42 km). The fact that distances across the entire range (1.5 to 42 km) produced MS_lim_ estimates to within 0.54 km/h make this new approach precise in addition to time–efficient, practical, and flexible.

The average treadmill speed selected by runners given 60 to 90% guard rails was 79% of the Table 1 MS_lim_ estimates. Self–selected treadmill speed represented steady state metabolism (Figures 2A, 3A) corresponding to 12.3 km/h. Highly trained female collegiate runners required to run at 90% of their MS_lim_ estimates ran faster than trained males but still achieved metabolic stability (Figures 2B, 3B), in accordance with race ability. The ΔV̇O_2_ from running minutes 4 through 5 fell between (34 to 66 mL/min) the strict threshold plateau recommended by some (<50 mL/min) (Astorino, 2009), but the null group outcomes of a zero slope were convincing (*p =* 0.31−0.88) and below the *a priori* slope range estimated to produce arguably indifferent drift (50 to 150 mL/min).

The inability of the highly trained runners to complete 5-minutes at 110% of their MS_lim_ estimate (roughly 3 km performance speed) was surprising. However, this could be the result of testing runners outside of their competitive season. If Table 1 estimates were optimistic, small estimation errors (Figure 1) might have been magnified. Since the 90% trial always preceded the 110% trial with a short rest between (5-min), it is reasonable that small over–estimation errors in MS_lim_ could have expedited fatigue at 110% (Azevedo et al., 2021; Iannetta et al., 2018).

One limitation to using the estimation equations in Table 1 is that the lower bound of performance times may still be too fast for some. For example, the slowest marathon time represented by our equations (Table 1) was ~3 h 50 minutes (MS_lim_ = 11.9 km/h), but more than half of all New York City Marathon finishers require ≥4.5 h (≤9.3 km/h) (Knechtle et al., 2021). Jack Daniels’ running tables (Daniels, 1979, 2014) include slower values, but we excluded any whereby the lower bound of the MS_lim_ estimate (i.e., 60%) was below ~7.2 km/h to avoid any overlap with walking speeds. In circumstances where runners are slower, one could estimate MS_lim_ as the quotient of mean marathon running speed (e.g., 9.3 km/h) (Knechtle et al., 2021) and 0.9, as it has been observed that marathon–length running events are contested at ~90% of MS_lim_ estimates (Jones et al., 2021). We may then estimate MS_lim_ for this runner as 10.33 km/h (i.e., 9.3/0.9). Any desired percentage of the MS_lim_ estimate from Table 1 can also be calculated, keeping in mind minimum speeds.

Daniels (1979, 2014) also acknowledges limitations to using the VDOT tables which would apply equally for our estimation equations derived from VDOT tables (Table 1). For example, when using one race performance to estimate another, estimations should be most reliable when achieved during periods of similar training status. Based on this caution, we required that all race times provided were achieved within the past year. Importantly, the same would be true for any estimate or measurement of MS_lim_ because seasonal changes in competitive fitness will inevitably alter fitness and performance. Daniels also points out that longer distances run outdoors in varied weather, over changing topography, or an occasional ‘bad day’ would create more uncertainty when extrapolating performance from one distance to the next (Daniels, 1979, 2014). To minimise this limitation, we recommend using the average Table 1 MS_lim_ estimate when more than one race distance is available.

Finally, the utility of our first approximation of CS from VDOT is strictly in estimating an upper limit for MS_lim_ in terms of the boundary for steady–state V̇O_2_. It does not substitute for definitive measurements of CS when true knowledge of CS is required (Hughson et al., 1984).

## Conclusion

Our results support using the equations in Table 1 in conjunction with recent times from 1.5 to 42 km races as an expedient method for selecting treadmill speeds that produce metabolic stability. The translation of a single race performance into an estimate of running speed producing reliable metabolic stability fills a meaningful gap in applied exercise physiology (Balasekaran et al., 2023; MacIntosh et al., 2021; Wolpern et al., 2015), one that can assist with exercise prescription, protocol standardisation, and reduce both time demands and participant burden. Our results empower sports scientists, clinicians, and coaches in settings where comprehensive metabolic testing is impractical or impossible. We encourage others to use and test our equations to more broadly understand their applications, limitations, and their relation to more traditional and empirical measures of metabolic stability (Jones et al., 2019).

## References

[cit0001] Astorino, T. A. (2009). Alterations in VO2max and the VO2 plateau with manipulation of sampling interval. *Clinical physiology and functional imaging*, *29*(1), 60–67. 10.1111/j.1475-097X.2008.00835.x 19125732

[cit0002] Azevedo, R. A, Forot, J., Iannetta, D., MacInnis, M. J., Millet, G. Y., & Murias, J. M. (2021). Slight power output manipulations around the maximal lactate steady state have a similar impact on fatigue in females and males. *Journal of Applied Physiology: Respiratory, Environmental and Exercise Physiology*, *130*(6), 1879–1892. 10.1152/japplphysiol.00892.2020 33914658

[cit0003] Balasekaran, G., Loh, M. K., Boey, P., & Ng, Y. C. (2023). Determination, measurement, and validation of maximal aerobic speed. *Scientific Reports*, *13*(1), 8006. 10.1038/s41598-023-31904-1 37198204 PMC10192137

[cit0004] Daniels, J. (1979). *Oxygen Power: Performance Tables for Distance Runners*. Daniels J and Gilbert J.

[cit0005] Daniels, J. (2014). *Daniels’ Running Formula*. Champaign, IL: Human Kinetics, Inc.

[cit0006] Daniels, J. T. (1985). A physiologist's view of running economy. *Medicine and Science in Sports and Exercise*, *17*(3), 332–338. 3894870

[cit0007] Dawson-Saunders, B., & Trapp, R. G. (1994). *Basic and Clinical Biostatistics*. Norwalk, CT: Appleton & Lange.

[cit0008] Goulding, R. P., Rossiter, H. B., Marwood, S., & Ferguson, C. (2021). Bioenergetic mechanisms linking V˙O_2_ kinetics and exercise tolerance. *Exercise and Sport Sciences Reviews*, *49*(4), 274–283. 10.1249/JES.0000000000000267 34547760 PMC8528340

[cit0009] Grassi, B., Porcelli, S., Salvadego, D., & Zoladz, J. A. (2011). Slow VO_2_ kinetics during moderate-intensity exercise as markers of lower metabolic stability and lower exercise tolerance. *European Journal of Applied Physiology and Occupational Physiology*, *111*(3), 345–355. 10.1007/s00421-010-1609-1 20821336

[cit0010] Hughson, R. L., Orok, C. J., & Staudt, L. E. (1984). A high velocity treadmill running test to assess endurance running potential. *International Journal of Sports Medicine (Stuttgart)*, *5*(1), 23–25. 10.1055/s-2008-1025875 6698679

[cit0011] Iannetta, D., Inglis, E. C., Fullerton, C., Passfield, L., & Murias, J. M. (2018). Metabolic and performance-related consequences of exercising at and slightly above MLSS. *Scandinavian Journal of Medicine and Science in Sports*, *28*(12), 2481–2493. 10.1111/sms.13280 30120803

[cit0012] Jamnick, N. A., Pettitt, R. W., Granata, C., Pyne, D. B., & Bishop, D. J. (2020). An examination and critique of current methods to determine exercise intensity. *Sports Medicine*, *50*(10), 1729–1756. 10.1007/s40279-020-01322-8 32729096

[cit0013] Jones, A. M., Burnley, M., Black, M. I., Poole, D. C., & Vanhatalo, A. (2019). The maximal metabolic steady state: Redefining the ‘gold standard’. *Physiological Reports*, *7*(10), e14098. 10.14814/phy2.14098 31124324 PMC6533178

[cit0014] Jones, A. M., Kirby, B. S., Clark, I. E., Rice, H. M., Fulkerson, E., Wylie, L. J., Wilkerson, D. P., Vanhatalo, A., & Wilkins, B. W. (2021). Physiological demands of running at 2-hour marathon race pace. *Journal of Applied Physiology: Respiratory, Environmental and Exercise Physiology*, *130*(2), 369–379. 10.1152/japplphysiol.00647.2020 33151776

[cit0015] Knechtle, B., McGrath, C., Goncerz, O., Villiger, E., Nikolaidis, P. T., Marcin, T., & Sousa, C. V. (2021). The role of environmental conditions on master marathon running performance in 1,280,557 finishers the ‘New York City Marathon’ From 1970 to 2019. *Frontiers in Physiology*, *12*, 665761. 10.3389/fphys.2021.665761 34079472 PMC8165243

[cit0016] Lansley, K. E., Dimenna, F. J., Bailey, S. J., & Jones, A. M. (2011). A ‘new’ method to normalize exercise intensity. *International Journal of Sports Medicine (Stuttgart)*, *32*(7), 535–541. 10.1055/s-0031-1273754 21563028

[cit0017] MacIntosh, B. R., Murias, J. M., Keir, D. A., & Weir, J. M. (2021). What is moderate to vigorous exercise intensity? *Frontiers in Physiology*, *12*, 682233. 10.3389/fphys.2021.682233 34630133 PMC8493117

[cit0018] Matthews, I. R., Heenan, L. J., Fisher, K. G., Flood, E. F., Wehrman, L. W., Kirby, B. S., & Wilkins, B. W. (2023). Identification of maximal steady-state metabolic rate by the change in muscle oxygen saturation. *Journal of Applied Physiology: Respiratory, Environmental and Exercise Physiology*, *134*(6), 1349–1358. 10.1152/japplphysiol.00706.2022 37078501

[cit0019] McKay, A. K. A., Stellingwerff, T., Smith, E. S., Martin, D. T., Mujika, I., Goosey-Tolfrey, V. L., Sheppard, J., & Burke, L.M. (2022). Defining training and performance caliber: A participant classification framework. *International Journal of Sports Physiology and Performance*, *17*(2), 317–331. 10.1123/ijspp.2021-0451 34965513

[cit0020] Padulo, J., Annino, G., Smith, L., Migliaccio, G. M., Camino, R., Tihanyi, J., & D’Ottavio, S. (2012). Uphill running at iso-efficiency speed. *International Journal of Sports Medicine (Stuttgart)*, *33*(10), 819–823. 10.1055/s-0032-1311588 22562739

[cit0021] Pettitt, R. W. (2016). Applying the critical speed concept to racing strategy and interval training prescription. *International Journal of Sports Physiology and Performance*, *11*(7), 842–847. 10.1123/ijspp.2016-0001 27197057

[cit0022] Pettitt, R. W., Clark, I. E., Ebner, S. M., Sedgeman, D. T., & Murray, S. R. (2013). Gas exchange threshold and VO2max testing for athletes: An update. *Journal of Strength and Conditioning Research*, *27*(2), 549–555. 10.1519/JSC.0b013e31825770d7 22531615

[cit0023] Poole, D. C., Burnley, M., Vanhatalo, A., Rossiter, H. B., & Jones, A. M. (2016). Critical power: An important fatigue threshold in exercise physiology. *Medicine and Science in Sports and Exercise*, *48*(11), 2320–2334. 10.1249/MSS.0000000000000939 27031742 PMC5070974

[cit0024] Reed, J. L., & Pipe, A. L. (2014). The talk test: A useful tool for prescribing and monitoring exercise intensity. *Current Opinion in Cardiology (London)*, *29*(5), 475–480. 10.1097/HCO.0000000000000097 25010379

[cit0025] Robergs, R. A., Dwyer, D., & Astorino, T. (2010). Recommendations for improved data processing from expired gas analysis indirect calorimetry. *Sports Medicine*, *40*(2), 95–111. 10.2165/11319670-000000000-00000 20092364

[cit0026] Scudamore, E. M., Barry, V. W., & Coons, J. M. (2018). An evaluation of time-trial-based predictions of VO2max and recommended training paces for collegiate and recreational runners. *Journal of Strength and Conditioning Research*, *32*(4), 1137–1143. 10.1519/JSC.0000000000001942 28426511

[cit0027] Wasserman, K., Stringer, W. W., Casaburi, R., Koike, A., & Cooper, C. B. (1994). Determination of the anaerobic threshold by gas exchange: Biochemical considerations, methodology and physiological effects. *Zeitschrift fuer Kardiologie*, *83*(3), 1–12. 7941654

[cit0028] Wolpern, A. E., Burgos, D. J., Janot, J. M., & Dalleck, L. C. (2015). Is a threshold-based model a superior method to the relative percent concept for establishing individual exercise intensity? A randomized controlled trial. *BMC Sports Science, Medicine and Rehabilitation*, *7*, 16. 10.1186/s13102-015-0011-z PMC4491229 26146564

